# Exploiting Activated Carbon Felt as a Selective Adsorbent for Removal of CO_2_ from Methane in a Pressurized Fixed-Bed Column

**DOI:** 10.1021/acsomega.6c00328

**Published:** 2026-03-26

**Authors:** Jimmy D. L. Moreno, Syed S. Shah, Ernesto A. Urquieta-González, Luís A. M. Ruotolo

**Affiliations:** † Department of Chemical Engineering, 67828 Federal University of São Carlos, Rod. Washington Luiz, km 235, São Carlos, SP 13565-905, Brazil; ‡ Research Center on Advanced Materials and Energy, 67828 Federal University of São Carlos, Rod. Washington Luiz, km 235, São Carlos, SP 13565-905, Brazil

## Abstract

This work investigates commercial activated carbon felt (ACF) as a selective adsorbent for CO_2_ separation from methane in pressurized fixed-bed columns, for potential use in off-shore natural gas purification. Pristine ACF was chemically modified, aiming to improve its adsorption capacity, followed by comprehensive characterization using TGA, XRD, FT-IR, N_2_ physisorption, SEM, and XPS to evaluate the textural properties and surface chemistry of the material. Breakthrough curves for CO_2_ uptake were obtained at 35 °C, up to 10 bar, for both the individual gases and binary CO_2_ and CH_4_ mixtures. The pristine ACF presented a high specific surface area (1946 m^2^ g^–1^), a microporous structure, and limited surface oxygen groups, resulting in an exceptional experimental CO_2_ adsorption capacity of 12.2 mmol g^–1^ at 10 bar and a CO_2_/CH_4_ selectivity ratio of 6.7, comparable to that of commercial zeolite 13X. Surface oxidation with nitric acid increased the quantity of oxygen groups, but severely degraded the textural properties, reducing adsorption performance. The results showed that the adsorption on ACF was primarily governed by the textural properties, with the pristine ACF outperforming several benchmark materials, including amino-MOFs and functionalized carbons. The findings highlighted commercial ACF as a promising low-cost and scalable adsorbent for natural gas decarbonization in pressure swing adsorption (PSA) systems.

## Graphical abstract

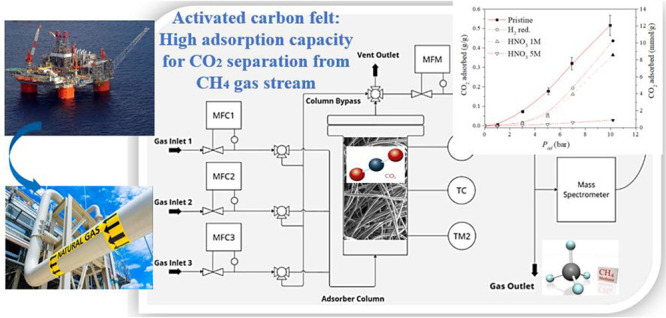

## 1 Introduction

The capture and storage of carbon dioxide (CO_2_) from various sources, including its removal from process streams for many valuable products, is an issue that remains at the forefront of research, due to the recognized greenhouse effect of CO_2_ and the associated environmental impacts.^1–3^ Given the current drive to diversify energy resources, considerable efforts have been made in developing decarbonization technologies for natural gas, together with advances in new renewable energy sources, aiming to reduce greenhouse gas emissions. This reduction will remain a priority in the coming decades, due to the emergence of new regulations in national legislations and the implementation of global international agreements.^4^ Although a range of processes can be used for natural gas purification (such as membrane or absorption methods, among others),^5–7^ adsorption still remains the most promising technique, with many adsorbents having been developed over the years.^8,9^

In this respect, the high CO_2_ content of natural gas (NG) produced from offshore platforms remains a challenging issue, especially for NG obtained from presalt underground marine deposits, where CO_2_ can account for a molar content of 30–50%.^10^ Long maritime transport distances and lack of space on offshore platforms currently hinders the commercial use of this NG, resulting in it being reinjected into the wells. This has led to a demand for new technological strategies to remove CO_2_ at offshore platforms, to obtain clean NG. Once the CO_2_ and other contaminants are removed, the NG consists almost entirely of CH_4_ at high purity, making its shipping economically viable. In addition, the CO_2_ removed from NG is suitable for reinjection into wells, supporting decarbonization efforts and mitigating environmental concerns.^11,12^

To address these challenges, a combined approach has been proposed, employing supersonic separation followed by adsorption, involving the development of new adsorbents.^12,13^ This strategy can achieve a low CO_2_ concentration (≤4%) in NG, ensuring both economic feasibility and compliance with NG transportation requirements.^14,15^ Although the supersonic step has proven effective for the rapid removal of water and other contaminants by means of condensation, it is not totally effective for CO_2_ removal.^11,12^ One strategy to resolve this drawback is to use compact supersonic separators to reduce the CO_2_ molar content by up to 20%,^13,16^ which is a realistic value to ensure economic feasibility, in combination with subsequent adsorption to achieve the required final CO_2_ concentration.

Adsorption is a well-recognized technology employed for CO_2_ separation, offering ease of operation, low energy consumption, environmental compatibility, cost-effectiveness, and recyclability.^17^ However, a challenge for the use of adsorption units on offshore platforms is the size of the adsorption column, making it necessary to identify new porous adsorbents with high capacity and selectivity for CO_2_ uptake. Among the various groups of materials used for adsorption, carbon-based adsorbents are particularly attractive, due to their high surface area, porosity, recyclability, and chemical properties that enable superior CO_2_ uptake and selectivity.^4,18,19^

Activated carbons are among the most promising adsorbents for CO_2_ adsorption, with advantages including wide commercial availability, low cost, high surface functionalization tailored for CO_2_ adsorption, fast adsorption kinetics, hydrophobic properties, high thermal and mechanical stability, and low energy consumption during regeneration.^20^ For the use of activated carbons in CO_2_ separation, it is essential that they should provide high levels of selectivity and adsorption capacity, which can be mainly attributed to the presence of electron-rich surface oxygen functional groups (SOGs) on the carbon, such as hydroxyl (R–OH), carbonyl (R–CO), and carboxyl (R–COOH) groups. These SOGs have been reported to enhance electrostatic interaction between the carbon surface and CO_2_ molecules, improving affinity and acting as adsorption sites for binding. In addition, SOGs preferentially favor adsorption of CO_2_, rather than CH_4_, which can also promote higher CO_2_/CH_4_ selectivity,^21–23^ due to π–π conjugation interactions that facilitate the uptake of CO_2_ molecules.^24^

Given the features of the adsorption mechanism, most commercial activated carbons do not fit the requirements for CO_2_ adsorption, since their industrial production typically employs activation with water vapor,^25^ which is unable to generate the high porosity and SOGs on the carbon surface that are required to provide adsorption sites and promote rapid kinetics. For this purpose, chemically activated carbons (CACs) produced using agents such as KOH and NaOH may be recommended, since this activation procedure can provide a variety of SOGs and high porosity associated with micropores and mesopores,^26,27^ leading to enhanced CO_2_ adsorption capability.

Over the past decades, considerable research effort has been made to investigate and modify activated carbons for CO_2_ adsorption, including the recent development of CACs suitable for use as pellets in adsorption columns. A major disadvantage of CACs is that they are often produced as fine powders, with subsequent pelletization requiring binders that can reduce the specific surface area by blocking access to adsorption sites within the pores.^28,29^ Since it is essential that adsorbents for use in industrial adsorption columns should be pelletized, significant progress is still needed in the development of activated carbons that can meet commercial requirements.

There has been growing interest in the development of CAC fibers,^30,31^ which is particularly relevant given the limitations of powdery materials. Aiming to meet engineering requirements, activated carbon felts (ACFs) have emerged as promising materials for use in adsorption columns. The well-defined pore structures of ACFs provide high capacity and rapid adsorption for specific compounds,^32^ together with homogeneous pore size distribution, large open-pore structure, and self-sustainable characteristics.^33^ These features make ACFs especially useful, when compared to conventional granular or powder activated carbons, as they can potentially eliminate the disadvantages of traditional pelletization processes. The hierarchical porous structure of ACFs has led to their investigation for use in applications such as gas adsorption,^33^ water purification,^34^ sound absorption,^35,36^ and electrode materials.^37,38^

Therefore, in this work, it was hypothesized that ACF would be a promising candidate for use in pressure swing adsorption (PSA) columns, where its high voidage would provide high permeability and facilitate mass transfer, while the presence of oxygen surface groups would improve CO_2_ uptake. The hierarchical porous structure, with the presence of large mesopores, would facilitate internal mass transfer to the adsorption sites, predominantly located within the micropores, resulting in faster adsorption kinetics. These features of ACF, combined with its high specific surface area (SSA) and the presence of electron-rich oxygen functional groups, would contribute to achieving a high CO_2_ adsorption capacity (χ, in L kg^–1^).

Irrespective of the nature of the adsorbent, PSA systems can be effective options for application in columns for industrial CO_2_ separation.^39,41^ Therefore, high-pressure adsorption isotherms for fixed-bed adsorbers are required to obtain design data that enable the establishment of reliable operational conditions, ensuring the feasibility of adsorption under real-world conditions.

This work explores the potential of using a commercially available ACF as an effective and low-cost carbon in PSA columns for the adsorption of CO_2_ from a CH_4_ stream, since these gases are the main components in NG produced at offshore Brazilian presalt oil platforms. Textural and structural analyses were used to characterize the pristine ACF and to understand the mechanism of CO_2_ adsorption. In addition, modification of the ACF surface under a hydrogen atmosphere or with nitric acid solution was performed to investigate the influence of SOGs on process efficacy.^42–45^ The uptake and kinetics for CO_2_ adsorption using the pristine and modified ACFs were assessed by means of breakthrough curves (BTCs), obtained with a PSA system, providing insights into the effects of surface modification and operational pressure conditions on adsorption performance. The BTCs enabled determination of the CO_2_ removal capacities and adsorption profiles, as well as the proposal of a kinetic model consistent with the experimental data, providing fundamental understanding and the engineering parameters necessary for scale-up and the design of operational units.^46^ In addition to the adsorption of pure CO_2_ and CH_4_ under high pressures, BTCs were also obtained using a model CO_2_ and CH_4_ NG mixture, for a preliminary evaluation of the selectivity toward CO_2_ adsorption.

## 2 Experimental Section

### 2.1 Materials and ACF Modifications

Commercial activated carbon felt with thickness of 1.8 mm and density of 53 kg m^–3^ (ACN-211-20, Kynol, Germany) was used as a potential CO_2_ adsorbent. This material was chosen based on its excellent textural properties, previously investigated in our research group.^38^ The ACF was cut into discs with diameter matching the internal diameter of the fixed-bed adsorption column.

The pristine ACF was submitted to different chemical treatments, to investigate the influence of surface chemistry on CO_2_ adsorption. Reduction with hydrogen was applied to reduce any oxygen surface groups present on the carbon surface. This was performed by the temperature-programmed reduction (TPR) technique, using an AutoChem II 2920 instrument (Micromeritics). The sample (50 mg) was first pretreated at 350 °C (with heating at a rate of 20 °C min^–1^) for 30 min, under an atmosphere of helium supplied at 30 mL min^–1^, followed by cooling to 50 °C. Subsequently, the sample was heated to 400 °C (at 10 °C min^–1^), under a 20 mL min^–1^ flow of 10% (v/v) H_2_/N_2_. The temperature selected for the H_2_ reduction considered the thermal stability of the material, indicated by the TGA profile.

Oxidation of the carbon surface using nitric acid was used to introduce SOGs. For this, the ACF samples were immersed in HNO_3_ (1 or 5 M), ultrasonicated for 30 min, and transferred to a reflux system consisting of a three-neck round-bottom flask placed on a heating mantle, where the solution was maintained at 100 °C for 4 h. Finally, the ACF was separated by filtration, washed with Milli-Q water until constant pH (6.9), and dried in an oven at 110 °C for 24 h.

### 2.2 ACF Characterizations

The pristine and modified ACFs were characterized by Fourier transform infrared (FT-IR) spectroscopy, X-ray diffraction (XRD), hydrogen temperature-programmed reduction (H_2_-TPR), scanning electron microscopy (SEM), thermogravimetric analysis (TGA), and X-ray photoelectron spectroscopy (XPS).

The TGA employed a TA Instruments Q600 analyzer (Artisan Technology Group), with heating of the sample from room temperature to 800 °C, at 10 °C min^–1^, under a 20 mL min^–1^ flow of N_2_. The thermogravimetric analyses were carried out to establish the temperatures for hydrogen treatment and regeneration of the adsorbent.

The textural properties of the activated carbon felt were determined by N_2_ adsorption/desorption measurements, performed with an ASAP 2420 instrument (Micromeritics). Before analysis, the materials were degassed for 10 h at 300 °C, under vacuum. The BET specific surface area (SSA_BET_) and total pore volume (*V*_T_) were determined using the Brunauer–Emmett–Teller (BET) equation, based on linearization of the isotherm in the relative pressure range from 0.05 to 0.30 (*P/P*_0_). The micropore volume was calculated using the Dubinin–Radushkevich (DR) method. The mean pore diameter was obtained directly from the experimental data, according to the NLDFT method.

XRD was performed using a Rigaku Multiflex diffractometer equipped with a Cu tube and Ni filter, operating with Cu Kα radiation (λ = 0.1542 nm), at 40 kV and 30 mA. The diffraction angles (2θ) were measured at a goniometer speed of 2° min^–1^, in the 2θ range from 5° to 70°.

FT-IR analyses employed a Bruker Vertex 70 spectrometer equipped with a platinum-diamond attenuated total reflectance (ATR) accessory. Spectra were recorded in the range 4000–400 cm^–1^, at a resolution of 4 cm^–1^, with accumulation of 64 scans.

XPS analyses were performed at the Brazilian National Nanotechnology Laboratory (LNNano/CNPEM), using a K-alpha XPS system (Thermo Scientific) with a monochromatic aluminum anode source. For survey data, the pass energy, dwell time, and energy step were set at 150, 10, and 1.000 eV, respectively, while settings of 50, 50, and 0.10 eV were used for high-resolution spectra. In both cases, the number of scans was 10 and the spot size was 300 μm.

### 2.3 Column Adsorption Methodology

The fixed-bed adsorption system, shown schematically in Figure 1, was designed and manufactured by Institut für Nichtklassische Chemie (Leipzig, Germany). The system was coupled to a ThermoStar gas analyzer consisting of an inlet arrangement, a PrismaPlus mass spectrometer (MS), a dry diaphragm vacuum pump, and a HiPace turbopump. The gas samples (CO_2_ and/or CH_4_) were fed into the analyzer through a quartz capillary and their concentrations were determined after calibration of the inlet gas concentration at the detection limit of 1 ppm.

**Figure 1:**
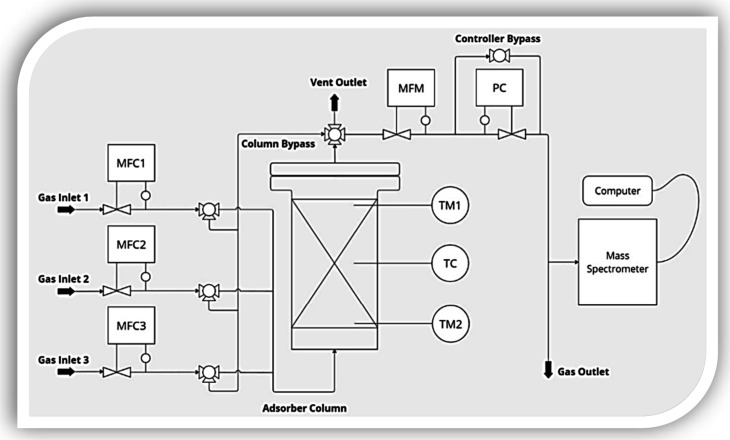
Schematic representation of the adsorption column and data collection system.

The adsorption column had a diameter (*D*) of 9 mm and length (*L*) of 99 mm. These dimensions (considering the selected ACF diameter) complied with the requirements for obtaining reliable breakthrough data, where the ideal setup should present *L/D* > 3 and *D/d* (fiber diameter) > 10,^47^ near-adiabatic conditions, and minimal dead volume (compared to the total column volume). The pressure drop, at below 0.02 bar, could be considered negligible.

The quantities of the gases adsorbed by the pristine ACF, at 35 °C, were obtained from the BTCs and plotted as a function of the applied pressure, for pure CO_2_ or CH_4_ (20 mL min^–1^), at five different pressures (1, 3, 5, 7, and 10 bar). For the modified ACF, the experiments were only conducted at 10 bar. All the experiments were performed in triplicate and the temperature was maintained constant at 35 °C during the experiments. It should be noted that prior to each experiment, the adsorbent was regenerated by applying a thermal treatment at 300 °C during 10 h, with a heating rate of 10 °C min^–1^. The experiment using a binary mixture of the gases considered a gas flow CO_2_/CH_4_ percentage ratio of 20/80, with CO_2_ and CH_4_ flow rates of 20 and 80 mL min^–1^, respectively, in accordance with the adopted separation strategy described in the Introduction section. In brief, an approach combining supersonic separation and adsorption has been proposed. Supersonic equipment would remove water, light hydrocarbons, part of the CO_2_, and other contaminants by condensation, until a CO_2_ molar content to 20%, resulting in a gas with 80% of methane. Then, the remaining CO_2_ would be removed by adsorption.

A four-step single fixed-bed PSA procedure was followed to obtain the isotherm points at high pressures, from breakthrough runs using the packed ACF bed: (1) pressurization (PR) using N_2_ until equilibration of the desired partial pressure; (2) adsorption (AD), with the gas flowing continuously through the ACF bed; (3) blowdown (BD), corresponding to the desorption step; and (4) purging (PG), carried out simultaneously with the PR step.

The amounts of adsorbed CO_2_ and CH_4_ were calculated from the BTCs obtained under the different operational conditions, using eq 1. The film mass transfer coefficients (*k*_f_, in m s^–1^) were also determined from the BTCs, using eq 2, according to the methodology described by Zafanelli et al.,^48^ based on the hypotheses and theoretical framework developed by Leyva-Ramos et al.,^49^ Leyva-Ramos and Geankoplis,^50^ and Ruthven and Helfferich.^51^ In eqs 1 and 2, *q*_i_^*^ is the amount adsorbed at equilibrium (mmol g^–1^ of adsorbent), *C*_0_ is the initial adsorbent concentration in the feed stream line (mmol L^–1^ of adsorbate), *Q* is the volumetric flow rate (L min^–1^), *V*_c_ is the column adsorption volume (L), *m*_ads_ is the adsorbent mass (g), ε is the column adsorption porosity, *d*_p_ is the fiber diameter (m), and *D*_m_ is molecular diffusivity (m^2^ s^–1^). *Sh*, *Sc*, and *Re* are the dimensionless Sherwood, Schmidt, and Reynolds numbers, respectively.

qi*=C0[Q∫01(1−CC0)dt]−εVcmads 1

$$Sh=\frac{d_{p}k_{f}}{D_{m}}=2.0+0.6Sc^{1/3}Re^{0.5}$$ 2

## 3 Results and Discussion

### 3.1 ACF Characterizations

The pristine and modified ACF samples were first characterized to enable understanding of the effects of the textural properties and surface chemistry on the adsorption uptake, since the number of adsorption sites is related to the surface area, while the adsorption mechanism may be influenced by the presence of surface oxygen groups.

SEM analyses were used to obtain information about the morphology of the material, before and after the introduction of surface modifications. Figure S1 shows low-magnification SEM images of the pristine ACF composed of smooth and tangled fibers, free of visible cracks, with average diameter of approximately 10 μm. Images acquired at higher magnification (Figure 2) showed that the surface of the ACF treated with nitric acid was significantly rougher and more irregular, compared to the unmodified pristine ACF, due to damage of the carbon framework during the acid treatment. Treatment with HNO_3_ also led to substantial weight loss, which could affect the CO_2_ adsorption capacity by collapse of the well-developed microporous channels, as highlighted in the inset of Figure 2c. After H_2_-reduction, the ACF showed a uniform increase of porosity, compared to the pristine ACF (Figure 2d), because the H_2_ treatment at 400 °C would facilitate the release of trapped volatile compounds, resulting in the formation of open blind pores.

**Figure 2:**
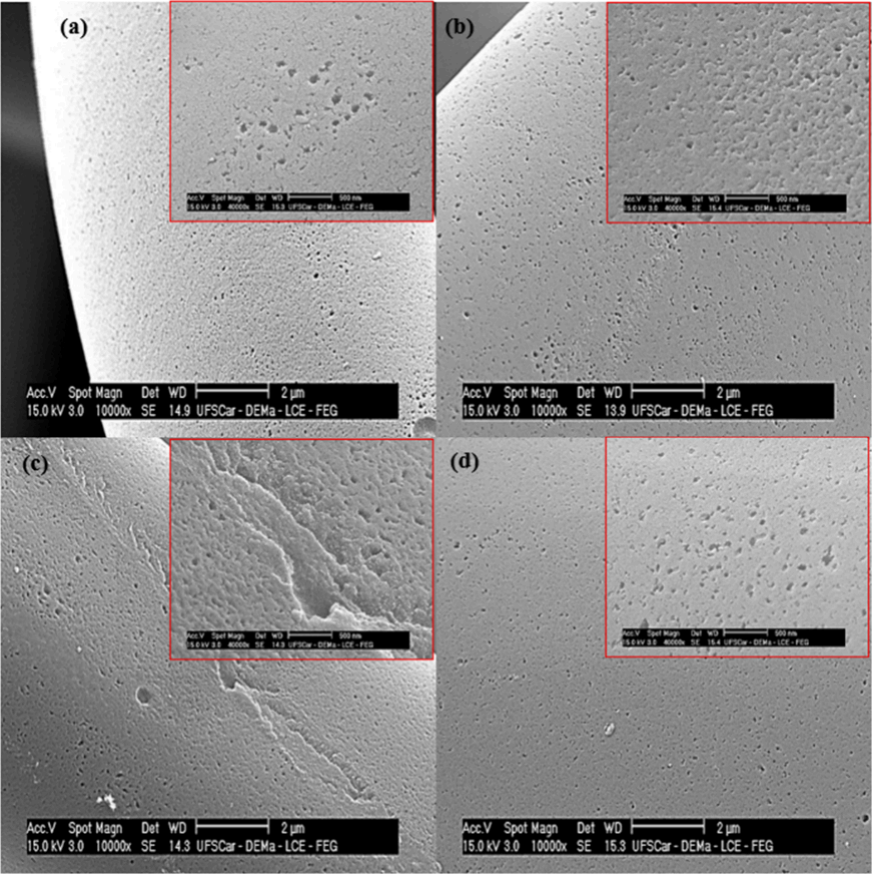
SEM images of the ACF samples: (a) pristine; (b) 1 M HNO_3_-treated; (c) 5 M HNO_3_-treated; (d) H_2_-reduced.

The TGA profiles of the pristine, HNO_3_-treated, and H_2_-reduced ACF samples are presented in Figure 3a. The pristine ACF showed considerably higher weight loss below 100 °C, compared to the HNO_3_-treated material. At temperatures higher than 500 °C, slower weight loss was observed for the H_2_-reduced ACF, compared to the other samples. The weight losses observed at temperatures below 110 °C could be attributed to the volatilization of light components (adsorbed gases) or the release of adsorbed water, while the drastic decreases in the range 500–625 °C were due to pyrolysis under a nitrogen atmosphere.^49^

**Figure 3:**
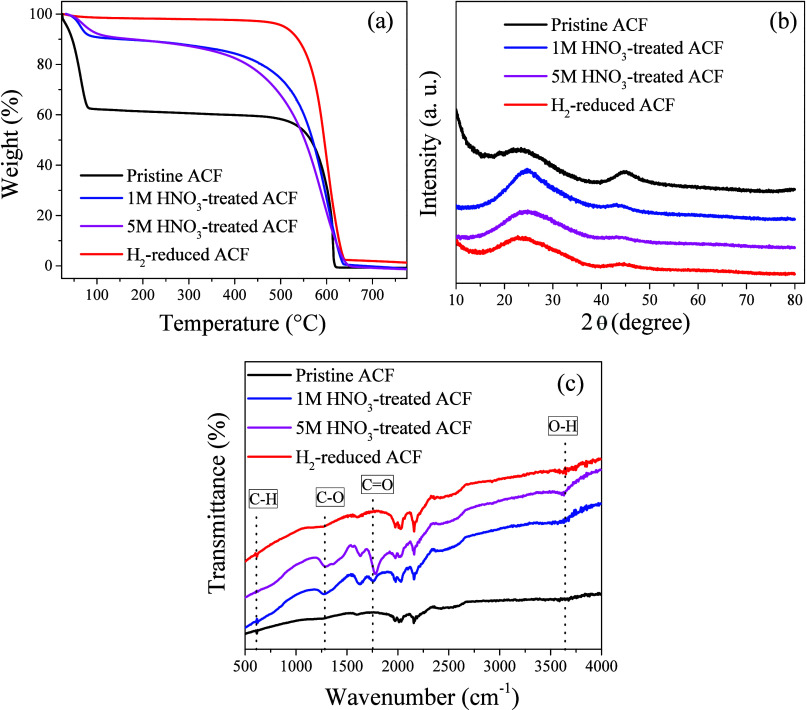
Characterization of the pristine and modified ACF: (a) TG profiles; (b) XRD patterns; (c) FT-IR spectra.

The faster weight losses for the nitric acid-treated samples suggested that the introduction of oxygen-containing functional groups on the surface, together with pyrolysis, could act in facilitating oxidation reactions. Considering the two HNO_3_-treated samples, the sample treated with 5 M HNO_3_ (which caused substantial mass loss during the harsh acid treatment) showed faster weight loss between 350 and 625 °C, compared to the ACF sample treated with 1 M HNO_3_. As shown in Figure 3a, at temperatures lower than 600 °C, the H_2_-reduced ACF sample exhibited the highest thermal stability, while at temperatures above 600 °C, most of the volatile components and pyrolytic substances were either decomposed or released, leading to a significant reduction in the weight loss rate. In contrast to the complete decomposition observed for the pristine ACF at about 625 °C, the HNO_3_-treated ACF samples retained small amounts of mass at that temperature. This residual mass was probably due to the oxygen-containing functional groups introduced during the acid treatment, which could form thermally stable compounds, consequently contributing to improved resistance at high temperatures.^43^

The X-ray diffractograms of the pristine and modified ACF are shown in Figure 3b. Before and after the treatment with nitric acid, the ACF presented typical characteristics of amorphous carbon, with weak diffraction peaks at 2θ of around 22° and 44°, indicative of graphitic carbon.^27^ No additional peaks appeared after modification of the pristine ACF, with the overall diffraction patterns remaining almost unchanged, suggesting that the treatments using nitric acid or H_2_-reduction did not significantly alter the ACF structure.^42^ Nevertheless, after the 1 M HNO_3_ treatment, the main diffraction peak appeared sharper and slightly shifted to the right, indicating an increase of the ACF crystallinity and a decrease in the interplanar spacing. This effect could be explained by the introduction of oxygen-containing functional groups during the modification process, which relaxed the lattice structure and promoted a more orderly arrangement.^44,45,52^

The FT-IR spectrum of the pristine ACF (Figure 3c) showed a band at around 600 cm^–1^, attributed to C–H stretching vibrations,^53^ while carbonyl absorption bands at 1276–1290 cm^–1^ and at 1700–1800 cm^–1^ could be attributed, respectively, to C–O groups and to C=O, COOH, or ester groups.^54,55^ Bands at 1900–2300 and 3600 cm^–1^ corresponded to CO_2_ and to hydroxyl group (O–H) stretching, respectively.^56^ These data indicated that the ACF contained hydroxyl and carbonyl/carboxylic functional groups able to actively adsorb CO_2_ molecules.^43,57^ As a result of the chemical treatment, the activated carbon felt contained multiple oxygen-containing groups (carbonyl groups, weak acid carboxyl groups, strong acid carboxyl groups, and phenolic hydroxyl groups).^57^ As shown in Figure 3c, the C–H group band was only absent for the 5 M HNO_3_-treated sample, while the C–O and C=O group bands were only observed in the spectra for the HNO_3_-treated samples, which also presented more evident O–H bands.

Considering that adsorption is a physical process in which molecules are adsorbed on a solid surface, the properties of that surface will define the capacity and effectiveness of a given material as a potential adsorbent for CO_2_. A suitable material should possess surface functional groups, high specific surface area (SSA), and an adequate pore system. High SSA is usually desirable, since it is related to the quantity of sites available for adsorption, consequently influencing the adsorption capacity. Microporous carbons with pores between 0.5 and 2.0 nm in diameter have been reported as the most suitable for CO_2_ adsorption,^58–61^ although it is also desirable to have the presence of mesopores, which can act as broad “avenues” to facilitate the diffusion of molecules to or from the micropores, enhancing the kinetics of the adsorption process. Based on these principles, the material studied in the present work was an activated carbon felt, which differs from particulate adsorbents in terms of the continuity of the solid phase, eliminating the requirement for pelletization binders that can significantly decrease the SSA of the adsorbent. Commercial carbon felts with very low SSA are easily available,^62^ so it was necessary to search for a material with the desired characteristics for adsorption, resulting in selection of the ACN-211-20 carbon felt, manufactured and supplied by Kynol, for use as a potential adsorbent for CO_2_ in this study.

The textural properties of the pristine ACN-211-20 ACF were characterized using N_2_ adsorption/desorption isotherms, shown in Figure 4. The isotherms for the modified ACF (Figure S2) followed the same pattern as for the pristine ACF. All the isotherms were type I, according to the IUPAC classification, indicating the predominance of a microporous structure, with an average pore diameter close to 1.0 nm and most of the pores having diameters smaller than 2 nm. The pore size distributions (insets in Figures 4 and S2) confirmed the microporous nature of the ACF. The textural properties of the pristine and modified materials are summarized in Table 1. In the case of the pristine and H_2_-reduced ACF samples, high SSA was also observed. The treatment with 1 M HNO_3_ led to a substantial decrease of SSA, although the microporous nature was preserved. When the severity of the acid treatment was increased, using 5 M HNO_3_, the fiber structure was compromised and the fibers became brittle. The textural properties deteriorated and this sample presented the lowest SSA.

**Figure 4:**
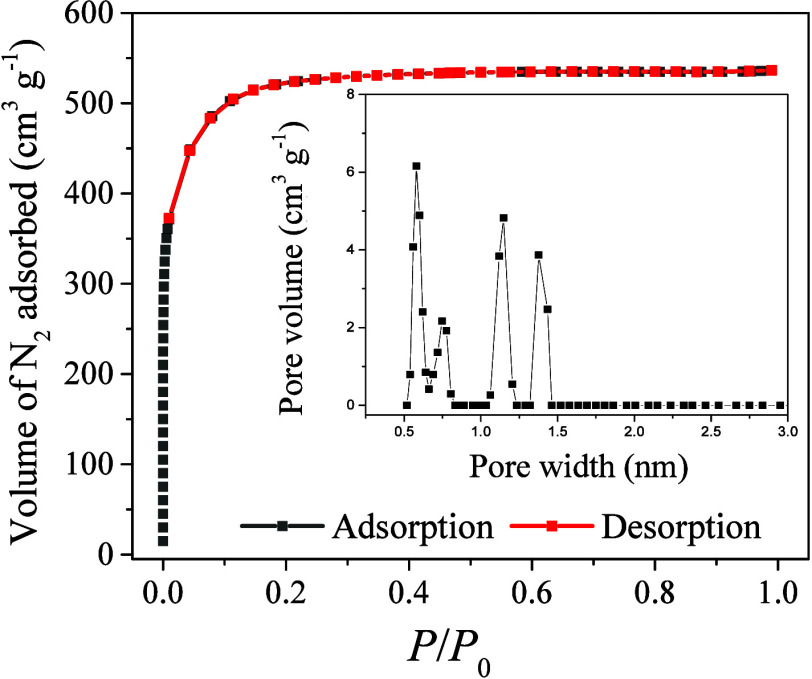
N_2_ adsorption/desorption isotherms for the pristine ACF.

**Table 1:** Textural Properties of the Pristine and Modified ACFs^a^

sample	*SSA*_BET_ (m^2^ g^–1^)	*V*_p_ (cm^3^ g^–1^)	*V*_mic_ (cm^3^ g^–1^)	*D*_p_ (nm)
pristine ACF	1946	0.829	0.823	0.93
H_2_-reduced	1934	0.790	0.767	0.95
1 M HNO_3_-treated	1295	0.597	0.551	1.01
5 M HNO_3_-treated	94.6	0.038	0.034	1.40

a *V*_p_, *V*_mic_, and *D*_p_ are the total volume of pores, the volume of micropores, and average pore diameter, respectively.

XPS analyses were performed to obtain further insights into the surface compositions of the pristine and modified ACFs, considering how the composition could affect the adsorption properties. The C 1s and O 1s XPS spectra are shown in Figure 5. The deconvoluted XPS C 1s spectrum, shown for the pristine ACF in Figure 5a, revealed peaks at 284.6, 285.3, and 289.4 eV, corresponding to C–C bonds, aliphatic C–H groups, and carboxyl (−COOH) groups, respectively.^63,64^ Similar features were observed for the H_2_-reduced and 1.0 M HNO_3_-treated samples (Figure 5b,c).

**Figure 5:**
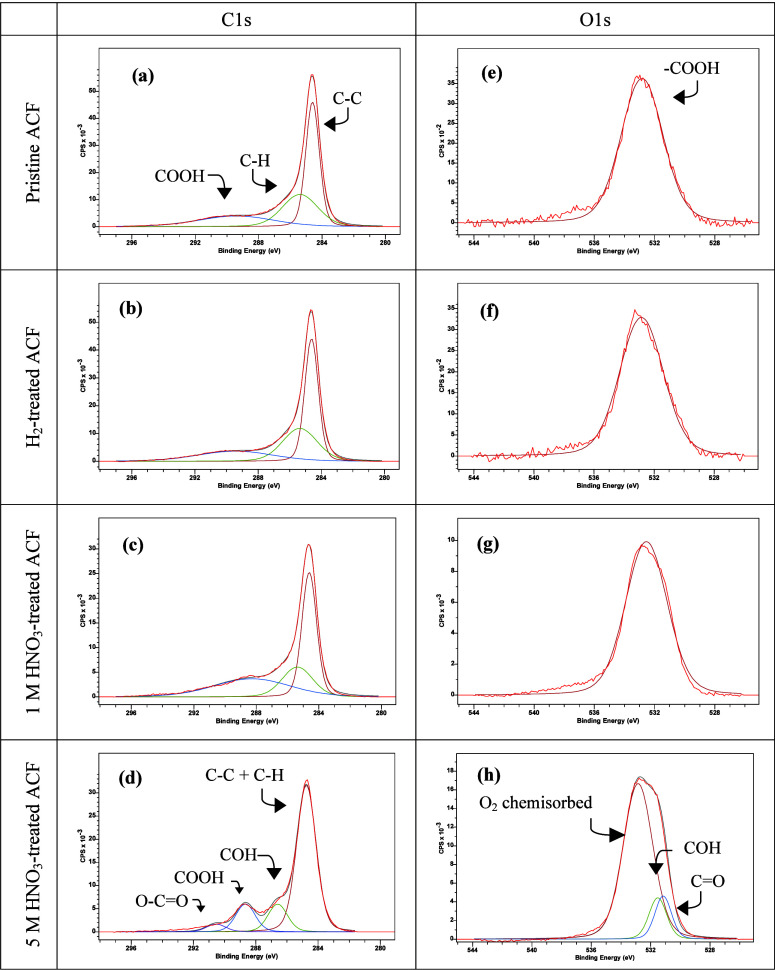
C 1s and O 1s XPS spectra of the pristine and modified ACF samples. (a) C 1s for pristine ACF; (b) C 1s for H_2_-treated ACF; (c) C 1s for 1 M HNO_3_-treated ACF; (d) C 1s 5 M HNO_3_-treated ACF; (e) O 1s for pristine ACF; (f) O 1s for H_2_-treated ACF; (g) O 1s for 1 M HNO_3_-treated ACF, and (h) O 1s 5 M HNO_3_-treated ACF.

The deconvolution shown in Figure 5d revealed a more oxidized surface and a significant change of surface oxygen functional groups (SOGs), with signals for carbon bonds and hydrocarbon groups at 284.7 eV, ether groups at 286.6 eV, carbonyl groups at 288.6 eV, and ester groups at 290.1 eV. The O 1s spectra shown in Figure 5e–g confirmed the major presence of the carboxylic group, with a peak at 532.8 eV for the pristine, H_2_-reduced, and 1.0 M HNO_3_-treated samples, while Figure 5h also shows an ether group peak at 530.4 eV.^63,64^

The elemental composition and surface group contents of the samples are summarized in Table 2, showing that the pristine ACF was composed of about 89% of carbon and hydrocarbon groups. After reduction with H_2_, there were almost no changes in the oxygen content or the contents and types of the different functional groups, indicating that the surface of the pristine ACF was poorly functionalized with SOGs, as confirmed by the O 1s/C 1s ratio, which was not higher than 0.12 for the pristine and H_2_-reduced samples. Compared with other activated carbons reported in the literature,^25,63,65^ the oxygen content in the pristine ACF could be considered low, indicating an opportunity for improvements by means of surface oxidation, as performed here by treatment of the pristine ACF with HNO_3_ solutions at different concentrations.

**Table 2:** Near-Surface Elemental Composition (Atomic %) and Surface Group Contents Obtained by XPS for the Pristine and Modified ACF Samples

sample	C 1s	O 1s	O 1s/C 1s	C–C	C–H	COOH	C=O	O–C=O	COH
pristine	89	11	0.12	0.47	0.31	0.22			
H_2_-reduced	95	5	0.12	0.47	0.32	0.21			
1 M HNO_3_-treated	66	34	0.51	0.44	0.22	0.34			
5 M HNO_3_-treated	57	43	0.77	0.70			0.13	0.04	0.13

The sample treated with 1 M HNO_3_ showed a 3.3-fold increase of the O 1s/C 1s ratio, with COOH (increase of about 62%) as the main detected surface group. The treatment with 5 M HNO_3_ appeared to have superoxidized the carbon surface (O 1s/C 1s = 0.77), leading to the generation of new surface oxygen groups. Considering that the adsorption capacity and selectivity could be improved by the interactions of SOGs with CO_2_ molecules,^22^ the modified ACFs were also evaluated using a pressurized fixed-bed adsorption column.

### 3.2 PSA Breakthrough Curves

The breakthrough curve is a valuable tool for evaluating the performance of adsorbents, simulating real operational conditions. Figure 6 shows the BTCs for the adsorption of CO_2_ and CH_4_ by the pristine ACF, evidencing the significant influence of pressure on the adsorption process, especially for CO_2_ uptake. The breakthrough time (*t*_b_) for CO_2_ adsorption ranged from 33 s (1 bar) to 5.4 min (10 bar), with t_b_ of 1.8 min at 5 bar, demonstrating an exponential increase of t_b_ as a function of the applied pressure (*P*_ad_; inset of Figure 6a). In contrast, the same *t*_b_ variation was not observed for methane adsorption (Figure 6b), suggesting weak selectivity of the pristine ACF toward CH_4_ adsorption, relative to CO_2_, as evidenced in Figure S3. Furthermore, for CO_2_ adsorption by the pristine AFC, the nonlinear increase of *t*_b_ as a function of *P*_ad_ was indicative of excellent adsorption capacity at 10 bar.

**Figure 6:**
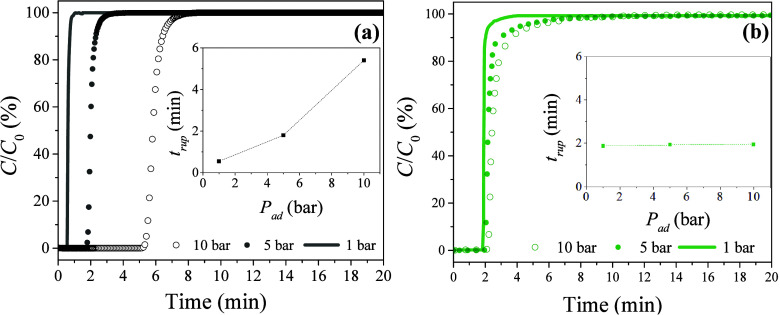
BTCs for adsorption of CO_2_ (a) and CH_4_ (b) at different *P*_ad_. Insets: Evolution of *t*_b_ according to the applied pressure. Experimental conditions: 35 °C; 20 mL min^–1^.

As shown in Figure 6, the BTC profiles for the two gases were quite similar. At lower *P*_ad_, the BTC profile after the breakthrough point (*C*/*C*_0_ ≅ 5%) was sharper, while a bulging effect only occurred close to the saturation zone. As *P*_ad_ increased, the inflections of the BTC profiles after the mass transfer zone (MTZ) became more pronounced, due to the deviations from ideal conditions related to the nondilution effect and volumetric flow of the gases, in addition to the fact that the process mostly occurred under pseudoequilibrium, favoring the apparent increase of the MTZ.^66–68^ It should also be noted that in all cases, the quasi-vertical shape of the MTZ for CO_2_ adsorption (Figure 6a) revealed very fast adsorption kinetics.

The effect of pressure on the film mass transfer coefficient was consistent with the BTC profile variations observed for CO_2_ (Figure 6a). Increase of the column pressure from 1 to 10 bar also led to proportional 10-fold decreases of *k*_f_ and *D*_m_ (Table 3). However, no sensitivity of *k*_f_ was observed for adsorption performed using the H_2_-reduced and acid-treated adsorbents (Tables S1 and S2).

**Table 3:** Breakthrough Time (*t*_b_), Adsorption Capacity (χ), Molecular Diffusivity (*D*_m_), and Film Mass Transfer Coefficient (*k*_f_) Values for CO_2_ Adsorption on the Pristine and Modified ACF Samples, under Different Pressures (*P*_ad_), at 35 °C

sample	*P*_ad_ (bar)	*t*_b_ (min)	χ (L kg^–1^)	*D*_m_ × 10^–6^ (m^2^ s^–1^)	*k*_f_ (m s^–1^)
	1	0.6	61	17.1	5.333
pristine	5	1.8	184	3.26	1.027
	10	5.4	552	1.54	0.490
H_2_-reduced	10	5.3	449	1.54	0.485
1 M HNO_3_-treated	10	3.8	163	1.54	0.484
5 M HNO_3_-treated	10	0.3	10	1.54	0.474

The highest CO_2_ uptake was obtained for the adsorption performed at 10 bar (Figure 6a), compared to 5 or 1 bar, as shown by the adsorption capacity (χ) values (Table 3). At 10 bar, the higher density of the adsorbate could increase the probability of intermolecular collisions, hindering mass transport from the gas phase to the adsorbent surface and making the diffusion pathway more tortuous, leading to lower *D*_m_ and *k*_f_ values. The decrease of *k*_f_ could be attributed not only to molecular competition, but also to a strong influence of the intrinsic affinity of the adsorbate for the adsorbent.^69,70^

Notably, the *k*_f_ values obtained in the present work were much higher than those reported in the literature for commercial activated carbons. For example, using theoretical correlations, Casas et al.^40^ obtained a *k*_f_ value of 0.001 m s^–1^ for a 50/50 mixture of CO_2_/H_2_, at 15 bar and 25 °C. Zafanelli et al.^48^ reported *k*_f_ of 0.116 m s^–1^ for adsorption using zeolite 4A, at 0 °C and 0.4 bar. A similar value of *k*_f_ (compared to the pristine ACF) was reported by Rahnema et al.^71^ for CO_2_ adsorption on a hollow fiber membrane, at 1 bar.

Modification of the adsorbent had a major effect on adsorption capacity, as shown by the adsorption capacity values (Table 3). For the H_2_-reduced ACF, the ∼23% decrease of χ was consistent with the textural and surface chemistry changes (Tables 1 and[Table tbl2]), with *SSA*_BET_, *V*_p_, and the O 1s/C 1s ratio only decreasing slightly after reduction under a hydrogen atmosphere. These results evidenced the importance of both textural properties and surface chemistry in determining the adsorption capacity.

The HNO_3_-treated adsorbents were severely impacted by the treatments, with the adsorbent modified using 1 M HNO_3_ showing a decrease of 2.4-fold for the CO_2_ adsorption capacity (χ) at 10 bar, despite the increase of SOGs revealed from the XPS analysis. Since the presence of SOGs enhances π–π conjugation and electrostatic interaction between the carbon surface and CO_2_ molecules,^24^ an adsorption capacity increase would be expected. However, this was negated by the deleterious effect of the acid treatment on the specific surface area and pore volume (Table 1), which decreased by 50 and 39%, respectively, consequently diminishing the quantity of adsorption sites and decreasing adsorption performance. This deleterious effect was even more pronounced for the ACF sample treated with 5 M HNO_3_, due to its superoxidized carbon structure (O 1s/C 1s = 0.77), which also reflected in a very low pore volume (0.038 cm^3^ g^–1^) and specific surface area (94.6 m^2^ g^–1^).

Table 4 shows the adsorption parameters obtained from the methane breakthrough curves. Under isothermal conditions, the molecular diffusivity of CH_4_ was less affected by pressure, compared to CO_2_ (Table S3), and could be considered almost constant.^72^ Accordingly, *k*_f_ was also less sensitive to pressure. However, the *k*_f_ values for CH_4_ adsorption were higher than those for CO_2_ (Table S1), due to the higher molecular diffusivity of CH_4_. It should be noted that significant alteration of *k*_f_ could occur for adsorption using mixtures of these gases. In addition, for all conditions, the CH_4_ adsorption capacity was lower than for CO_2_ (Figure 6).

**Table 4:** Breakthrough Time (*t*_b_), Adsorption Capacity (χ), Molecular Diffusivity (*D*_m_), and Film Mass Transfer Coefficient (*k*_f_) Values for CH_4_ Adsorption on Pristine ACF, under Different Pressures (*P*_ad_), at 35 °C

sample	*P*_ad_ (bar)	*t*_b_ (min)	χ (L kg^–1^)	*D*_*m*_ x 10^–6^ (m^2^ s^–1^)	*k*_*f*_ (m s^–1^)
	1	1.8	45.9	24.3	7.014
pristine ACF	5	1.9	48.5		6.108
	10	2.1	79.1		5.853

The amount of CO_2_ adsorbed according to the applied pressure is shown in Figure 7a, where the solid symbols are the data from the BTC experiments, while the open symbols and dotted lines represent the CO_2_ uptake estimated using the Aranovich-Donohue-Sips (ADS) model,^73,74^ with 10 bar as a reference pressure. The error bars became more evident as the pressure increased, due to compression of the flexible fibers of the ACF, which affected the column porosity and volume. Therefore, for reliable comparison of adsorption at different applied pressures, the column was carefully packed with ACF to ensure constant ε and *V*_c_ (eq 1).

**Figure 7:**
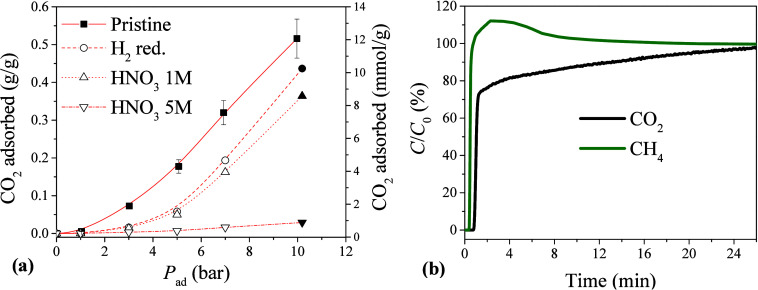
(a) CO_2_ adsorption, as a function of pressure, for the pristine and modified ACFs. (b) BTC profiles for CO_2_ and CH_4_ adsorption from a binary mixture (80% CH_4_/20% CO_2_, v/v), using the pristine ACF. Experimental conditions: 10 bar; 35 °C.

At low pressures, CO_2_ adsorption on the ACF increased nonlinearly with pressure, indicating that the process was dominated by physisorption, although chemisorption could also have contributed to the adsorption mechanism, and that the active sites had not yet become saturated. As the pressure approached and exceeded 10 bar, the adsorption curves showed slight leveling off, which according to the ADS model suggested that the microporous domains of the ACF were approaching saturation and that multilayer adsorption could occur on the external surface or in the wider pores.^75^ The good agreement between the experimental data and the ADS model, over the entire pressure range, confirmed the suitability of this model for describing adsorption on a heterogeneous carbon surface. This behavior reinforced that the mechanism of adsorption of CO_2_ on ACF was mainly governed by physical interactions that were enhanced under high-pressure conditions, consistent with the high surface area and microporous texture of the material.

According to Figure 7, the maximum adsorption capacity of the pristine ACF was 12.2 mmol CO_2_ g^–1^. Comparison of this value with the results reported by Gelles et al.^76^ for CO_2_ adsorption using different adsorbents revealed the potential of the ACF as an adsorbent for CO_2_. For example, the CO_2_ uptake capacity of the pristine ACF at 35 °C and 10 bar was significantly superior to the capacities achieved using adsorbents such as amino-impregnated MOF ZIF-8 (2.0 mmol CO_2_ g^–1^, at 5 bar and 45 °C),^77^ UIO-66 (4.6 mmol CO_2_ g^–1^, at 9.8 bar and 23 °C),^78^ HKUST-1 amino-MOF (2.3 mmol CO_2_ g^–1^, at 5 bar and 45 °C),^77^ and amino-functionalized AC (5.63 mmol CO_2_ g^–1^, at 0.66 bar and 50 °C).^79^

For all the samples, the CO_2_ adsorption capacity increased markedly with pressure (Figure 7), although the magnitude of the increase was strongly dependent on the surface treatment. The pristine ACF exhibited the highest uptake over the entire pressure range, followed by the H_2_-reduced sample, while the 5 M HNO_3_-treated material presented significantly lower adsorption capacity, especially at pressures higher than 4–5 bar. The lower performance of the acid-treated ACFs was consistent with the losses of surface area and pore volume caused by damage of the carbon framework, as discussed previously. The H_2_-reduced sample showed a similar pressure dependence, but with a smaller loss of capacity, which was probably due to partial removal of the oxygenated sites that contributed to CO_2_ adsorption. These results confirmed that both textural integrity and surface chemistry were critical in influencing CO_2_ adsorption under pressurized conditions.

In summary, analysis of the BTCs for single-component adsorption revealed that the pristine ACF exhibited higher CO_2_ uptake and longer breakthrough times at elevated pressures, while CH_4_ adsorption was less sensitive to pressure, with *k*_f_ decreasing at higher pressure, due to molecular competition, but remaining unaffected by the surface modifications.

Under real conditions for the separation of CO_2_ from NG, the presence of other molecules will affect mass transfer and lead to competition for adsorption sites. Therefore, a preliminary evaluation was made of the BTCs for a binary mixture, to elucidate the selectivity of the pristine ACF toward CO_2_ uptake at high pressures. The BTC profiles were obtained for a binary mixture of CO_2_ (20%) and CH_4_ (80%), which would be representative of NG leaving a prior supersonic separation unit at an offshore platform, as an example of an application under near-real conditions. The pristine ACF was chosen to adsorb CO_2_ from the binary mixture containing CH_4_.

The BTCs for uptake of CO_2_ and CH_4_ (Figure 7b) revealed higher affinity and superior adsorption capacity for CO_2_, with a CO_2_/CH_4_ selectivity ratio of 6.7 achieved after the saturation point (*C*/*C*_0_ ≅ 95%). The *C*/*C*_0_ values over 100% observed in the CH_4_ profile could be attributed to strong CO_2_ adsorption and a narrow mass transfer zone for CH_4_, leading to fast desorption of CH_4_ (the weakly adsorbed species), considering the advance of the CO_2_ adsorption front.^80^ The selectivity ratio obtained using the pristine ACF at 10 bar could be considered highly competitive, when compared to the selectivity ratio of 6.9 reported for the commercial 13X zeolite that is commonly employed for CO_2_ separation.^81^

## 4 Conclusions

Pristine activated carbon felt (ACF) was demonstrated to have remarkable potential as a selective and high-capacity adsorbent for CO_2_ separation under pressurized fixed-bed conditions. The CO_2_ uptake of 12.2 mmol g^–1^ at 10 bar, together with a competitive CO_2_/CH_4_ selectivity ratio of 6.7, represented performance rivaling or surpassing that of state-of-art adsorbents such as amino-MOFs and commercial zeolites. Although surface oxidation introduced additional oxygen groups, this treatment compromised the textural properties, leading to poor adsorption capacity. Reduction in a hydrogen atmosphere had only a minor effect, confirming that adsorption was predominantly controlled by textural properties, rather than surface chemistry. The results showed that the low-cost pristine ACF is a candidate for scalable applications in pressure swing adsorption systems, particularly in offshore natural gas decarbonization units, where compact operation areas and high-performance adsorbents are required. Future work should focus on optimizing functionalization strategies that preserve the unique textural features of ACF, while enhancing specific interactions with CO_2_.

## Supplementary Material



## Notes

Mass transfer and hydrodynamic parameters, SEM images, N_2_ adsorption/desorption isotherms, and Figure 6b magnification (PDF)
