# Supplementary material for: Exploiting Activated Carbon Felt as a Selective Adsorbent for Removal of CO2 from Methane in a Pressurized Fixed-Bed Column
Source: ACS Omega. 2026 Mar 26;11(13):21123–34. doi: 10.1021/acsomega.6c00328 (PMC13063077; doi:10.1021/acsomega.6c00328)
Supplement: Supplementary file 1 [file ao6c00328_si_001.pdf]

SUPPORTING INFORMATION

Exploiting activated carbon felt as a selective adsorbent for removal of CO_2 from methane in a pressurized fixed-bed column

Jimmy D. L. Moreno[a,b*], Syed S. Shah[a,b], Ernesto A. Urquieta-González[a,b], Luís A. M. Ruotolo[a*]

[a] *Department of Chemical Engineering, Federal University of São Carlos, Rod. Washington Luiz, km 235, São Carlos, SP, 13565-905, Brazil*

[b] *Research Center on Advanced Materials and Energy, Federal University of São Carlos, Rod. Washington Luiz, km 235, São Carlos, SP, 13565-905, Brazil*

Table S1. Linear velocity (*v*), molecular diffusivity (D_m), Reynolds number (*Re*), Schmidt number (*Sc*), and film mass transfer coefficient (k_f) values for CO_2 adsorption using the pristine ACF.

P (bar)	v x 10^{-3} (m s^{-1})	D_m x 10^{-6} (m^2 s^{-1})	*Re*	*Sc*	k_f (m s^{-1})
1.04	5.24	17.1	5.57	0.49	5.33
3.04	1.79	5.64	5.55	0.52	1.77
5.07	1.07	3.26	5.54	0.53	1.03
6.93	0.79	2.30	5.53	0..55	0.73
9.97	0.55	1.54	5.53	0.58	0.49

Table S2. Linear velocity (*v*), molecular diffusivity (D_m), Reynolds number (*Re*), Schmidt number (*Sc*), and film mass transfer coefficient (k_f) values for CO_2 adsorption using the 5 M HNO_3-treated ACF.

P (bar)	v x 10^{-3} (m s^{-1})	D_m x 10^{-6} (m^2 s^{-1})	*Re*	*Sc* x 10^{-1}	k_f (m s^{-1})
10.02	0.523	1.54	5.33	3.15	0.47

Table S3. Linear velocity (*v*), molecular diffusivity (D_m), Reynolds number (*Re*), Schmidt number (*Sc*), and film mass transfer coefficient (k_f) values for CH_4 adsorption using the pristine ACF.

P (bar)	v x 10^{-3} (m s^{-1})	D_m x 10^{-6} (m^2 s^{-1})	*Re*	*Sc*	k_f (m s^{-1})
0.99	5.24	24.3	2.69	0.72	7.01
2.97	1.75	24.3	2.69	0.24	6.34
5.05	1.03	24.4	2.69	0.14	6.11
7.00	0.74	24.4	2.69	0.10	5.98
9.94	0.52	24.3	2.69	0.72	5.85

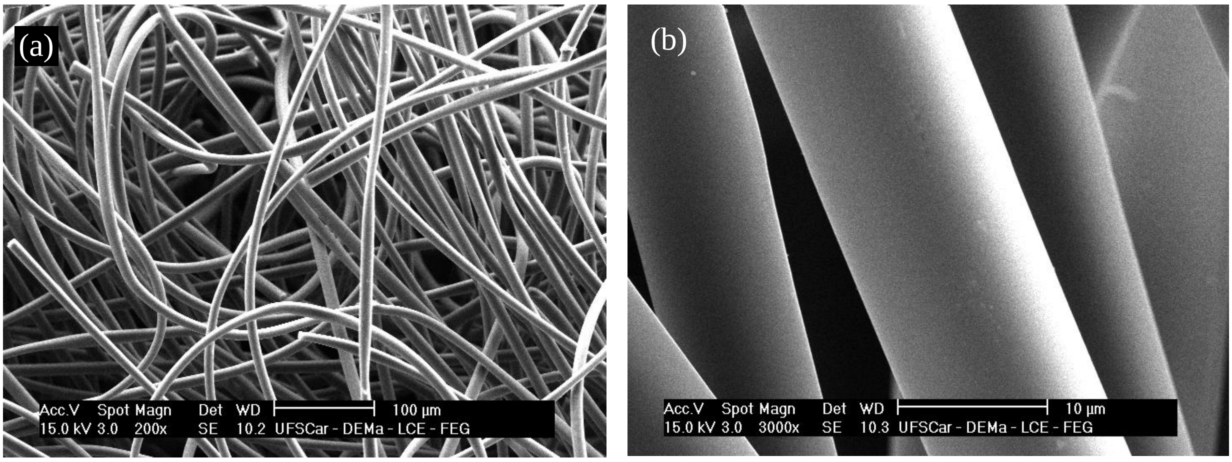

Figure S1. SEM images of the pristine ACF at low **(a)** and high **(b)** magnifications.

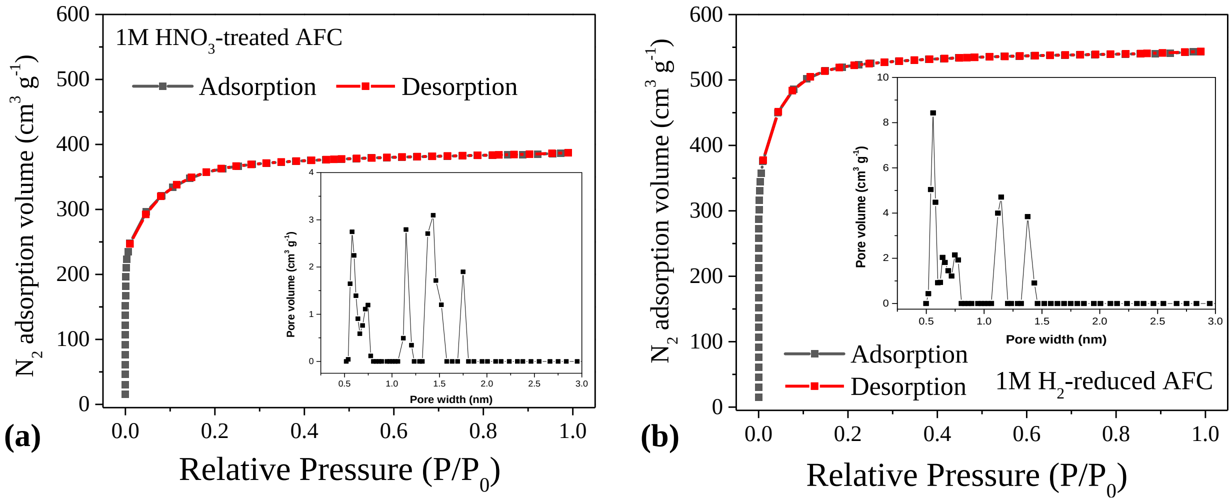

Figure S2. N_2 adsorption/desorption isotherms for the modified ACF: **(a)** 1 M HNO_3-treated ACF and **(b)** H_2-reduced ACF.

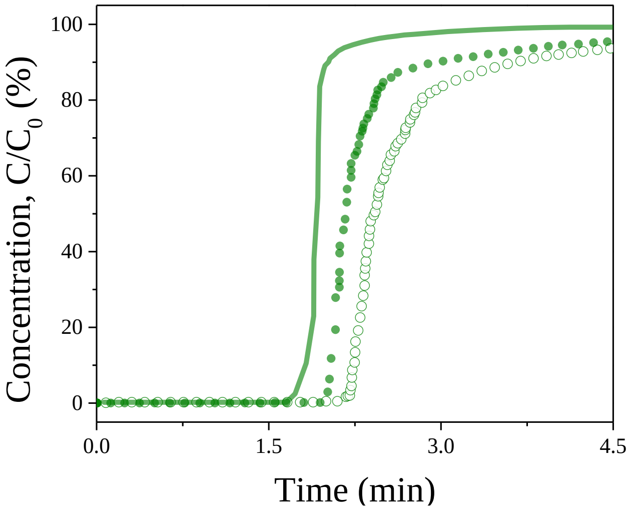

Figure S3. Magnification of Fig. 6b for CH_4 adsorption using the pristine ACF.
